# Cellular Components of the Tumor Environment in Gliomas—What Do We Know Today?

**DOI:** 10.3390/biomedicines12010014

**Published:** 2023-12-20

**Authors:** Reinhold Nafe, Elke Hattingen

**Affiliations:** Department of Neuroradiology, Clinics of Johann Wolfgang Goethe-University, Schleusenweg 2-16, D-60528 Frankfurt am Main, Germany; hattingen@med.uni-frankfurt.de

**Keywords:** gliomas, tumor environment, lymphocytes, tumor-associated macrophages, myeloid-derived suppressor cells, dendritic cells, cancer-associated fibroblasts, neutrophils, neurons, astrocytes

## Abstract

A generation ago, the molecular properties of tumor cells were the focus of scientific interest in oncology research. Since then, it has become increasingly apparent that the tumor environment (TEM), whose major components are non-neoplastic cell types, is also of utmost importance for our understanding of tumor growth, maintenance and resistance. In this review, we present the current knowledge concerning all cellular components within the TEM in gliomas, focusing on their molecular properties, expression patterns and influence on the biological behavior of gliomas. Insight into the TEM of gliomas has expanded considerably in recent years, including many aspects that previously received only marginal attention, such as the phenomenon of phagocytosis of glioma cells by macrophages and the role of the thyroid-stimulating hormone on glioma growth. We also discuss other topics such as the migration of lymphocytes into the tumor, phenotypic similarities between chemoresistant glioma cells and stem cells, and new clinical approaches with immunotherapies involving the cells of TEM.

## 1. Introduction

As research into the tumor environment (TEM) in gliomas has increased, tumor-associated macrophages (TAM) have become the cell type of greatest scientific interest in recent years, and there has been steady progress in understanding the subtypes of these cells and their contribution to tumor progression or even inhibition of tumor growth. Another advance is the increasing recognition that all other cell types within the TEM play important roles in the biological behavior of gliomas. Therefore, our review of the cellular components of TEM starts with the role of lymphocytes and new insights into the migration of T cells or even the role of B cells. The next important topics are new insights into microglia, subtypes of myeloid cells and glioma stem cells, with the current prevailing finding that these tumor stem cells do not represent a steady state during tumor progression but undergo changes in their phenotype. Neutrophils, mast cells and cancer-associated fibroblasts have also become increasingly important in glioma research. Dendritic cells are not present in the healthy human brain parenchyma, but they serve a variety of functions in gliomas. Finally, we discuss effects of non-neoplastic neuronal and glial cells on gliomas, in particular, recent findings on the special interaction between astrocytes and glioma cells via extracellular vesicles, gap junctions and tunneling nanotubes.

## 2. Lymphocytes

Compared to myeloid cells, lymphocytes are much less numerous in gliomas, with an estimated frequency of 1–5% of all cellular tumor components [1]. However, in contrast to their low numbers, they have an outstanding importance for the understanding of the tumor biology of gliomas. Most lymphoid cells in glioma tissue are CD8+ cells, whereas CD4+ “helper” cells are less abundant and the majority of these T cells lack antitumor activity. Moreover, a subset of CD4+ cells termed “T regulatory cells” or “Tregs” that express forkhead box protein 3 (FOXP3+) exhibit immunosuppressive activity with tumor-promoting properties [1,2,3]. Investigation of the divergent roles of T cells in gliomas, some of which exhibit tumor-promoting and tumor-inhibiting effects, begins with pathways regulating T cell invasion of glioma tissue. An experimental model of low-grade optic gliomas with mutation of neurofibromin 1 (NF1) showed increased expression of chemokine C-C ligand 5 (CCL5), along with other chemokines responsible for tumor growth, and increased attraction of T cells and microglia into tumor tissue [4]. An in vitro study of the expression of various chemokines in human glioblastoma cells revealed enhanced attraction of Tregs due to increased expression of the chemokine CCL22, whereas other tumor-derived chemokines showed no significant effect on Treg migration. Of note, blocking CCL22 expression did not completely eradicate Treg migration, and the authors address the need to identify other factors for T cell recruitment [5]. It is likely that disruption of the blood–brain barrier (BBB) is one of these factors, as glioma cells experimentally lead to the displacement of astrocytic end feet from endothelia and vascular smooth muscle cells, resulting in focal BBB disruption. In addition, these invasive glioma cells have been shown to control vascular tone through the calcium-dependent release of potassium, suggesting that glioma cells are capable of causing either constriction or dilation of tumor vessels, facilitating invasion and migration [6]. In addition, another pathway to recruit T cells to the glioma TME is the lymphatic system, with functional lymphatic vessels lining the dural sinus and connecting to the deep cervical lymph nodes [7]. This system is part of the recently discovered glymphatic system, which is considered an additional pathway of lymphatic drainage. The glymphatic system allows for the movement of CSF from the subarachnoid space along the periarterial space, where it mixes with the interstitial fluid within the brain parenchyma. However, the pathophysiological role of these lymphatic systems in gliomas has yet to be elucidated [7,8,9]. Regarding T cell migration, it has been confirmed in a *mouse* model that ectopic application of vascular endothelial growth factor C (VEGF-C) stimulates the migration of cytotoxic CD8+ T cells from deep neck lymph nodes to the glioma TME, leading to a long-lasting antitumor memory response of T cells. Song and colleagues interpreted the results as evidence for the possibility of recruiting cytotoxic T cells from cervical lymph nodes using VEGF-C as a potential therapeutic approach [10].

**Figure 1:**
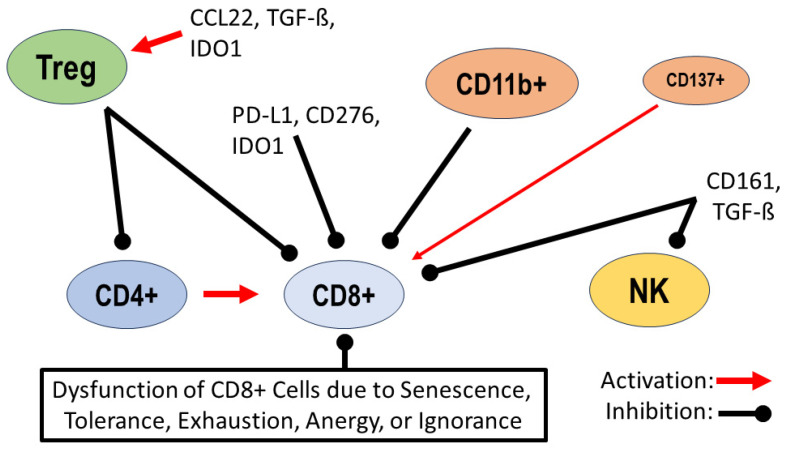
Dysregulation of lymphocytes in the tumor environment: activation of regulatory T cells (Tregs) by the chemokine CCL22, transforming growth factor ß (TGF-ß) and indoleamine 2,3-dioxygenase (IDO1); inhibition of migration and activity of CD4+ helper T cells and CD8+ effector T cells by Tregs. Inhibition of CD8+ cells by checkpoint proteins such as programmed cell death ligand 1 (PD-L1), B7 homolog 3 (CD276) and indoleamine 2,3-dioxygenase (IDO1). B cells expressing CD11b+ are inhibitors of CD8+ cells, and a minority of B cells expressing CD137+ support the antitumor activity of CD8+ cells; natural killer receptor protein 1 (CD161) and transforming growth factor ß (TGF-ß) inhibit the activity of natural killer (NK) cells and CD8+ cells. Additional dysfunction of CD8+ cells due to senescence, tolerance, exhaustion, anergy or ignorance [1,2,3,4,11,12,13,14,15,24,25,33,34,35,36,37].

In general, three main mechanisms can trigger T cell dysfunction in gliomas. These mechanisms are as follows: (1) the dysregulation of the expression of specific receptors and immune checkpoint proteins, leading to altered T cell differentiation and activity; (2) alterations in the recruitment and activity of major T cell subtypes such as Tregs and natural killer cells (NKs); (3) alterations in the interplay between T cells and other cell types such as microglia and macrophages (further discussed in the following chapter). One of the best characterized checkpoint proteins is programmed cell death ligand 1 (PD-L1), which is known to be expressed as a transmembrane protein on many peripheral cells such as T cells, B cells and monocytes, as well as in many tumor types. The extent of expression in high-grade and even low-grade gliomas is variable, with many tumors showing low expression, while tumors with marked expression exhibit an immunosuppressive environment due to the blockage of T cell differentiation and inhibition of CD8+ cell cytotoxic T cell activity [1,11,12]. Specifically, in low-grade gliomas, marked PD-L1 expression has been observed in a subset of cases that occur independently of the mutation status of the “*rapid accelerated fibrosarcoma B gene*” (*BRAF gene*). It is suggested that marked PD-L1 expression in low-grade gliomas represents a mechanism of tumor immune evasion that is independent of tumor mutational status [12]. Other checkpoint proteins of importance in glioma biology include the immunomodulator B7-H3 (CD276) and the tryptophan-degrading enzyme indoleamine-2,3-dioxygenase (IDO1). CD276 belongs to the immunomodulatory B7 family and is overexpressed in a variety of tumors, including gliomas. Guo and colleagues reported considerable expression of CD276 in high-grade gliomas, which has led to trials of antibody- and CAR-T cell-based immunotherapeutic approaches [13]. However, CD276 and its exact downstream cascade are still under investigation, and divergent results have been obtained for CD276 on whether it acts as a co-stimulator or co-inhibitor of T cell-mediated immune responses in different tumor types [13,14]. IDO1 shows only a weak expression in the adult CNS but an enhanced expression in many tumors such as gliomas. Immunosuppression by IDO1 occurs due to the degradation of tryptophan in the tumor environment. Initial success with IDO1 inhibitors has been achieved in phase 1 trials for immunotherapy of glioblastoma and experimentally in combination with radiotherapy and tryptophan substitution [15,16]. Thymocyte selection-associated high-mobility group box protein (TOX) is a DNA-binding protein involved in the regulation of immune cell development, including T cells. TOX expression is significantly reduced in high-grade gliomas compared with low-grade gliomas. TOX expression correlates significantly with longer overall patient survival and inversely with T cell and macrophage invasion. These results support the notion that high TOX expression contributes to an antitumor immunological environment in gliomas [17]. In gliomas with a mutation of the *isocitrate dehydrogenase gene* (IDH-mutation), decreased expression of the T cell-attracting chemokines CXCL9 and CXCL10 was found, resulting in reduced migration of CD8+ T cells to tumors in vitro. In vitro application of the oncometabolite α-hydroxyglutarate (α-HG), typical of IDH-mutated gliomas, confirmed its direct effect on reduced expression of the chemokines CXCL9 and CXCL10 and thus on reduced migration of CD8+ T cells [18]. Tregs are able to downregulate other immune cells, including CD4+, CD8+ and B cells, through cell-to-cell contact and even the secretion of various cytokines such as interleukin-10. In many tumor types, including gliomas, the T cell population has a higher percentage of Tregs than healthy controls, resulting in an immunosuppressive environment due to the suppression of other immune cells. For the activation of Tregs, transforming growth factor ß (TGF-ß) in particular is an important factor that promotes the formation of new Tregs and also the recruitment of existing Tregs. The chemokines CCL22 and IDO1 are other factors known to promote recruitment and activation of Tregs in gliomas (Figure 1) [1,19,20]. Natural killer (NK) cells can immediately initiate lysis of malignant or infected cells. The extent of this effect can be determined by altered expression of inhibitory or stimulatory receptors. In gliomas, an important receptor is natural killer receptor protein 1 (CD161), which is encoded by the *KLRB1 gene* (“*Killer Cell Lectin Like Receptor B1 gene*”) and acts as a suppressor of T cell cytotoxicity and NK cell activity. In a cohort of 313 glioma patients, Di and colleagues reported an increased expression of CD161 in glioblastomas and in IDH-mutated gliomas, and high CD161 expression inversely correlated with overall patient survival [21]. Increased activity of the *KLRB1 gene* has been described in many human cancers, overexpression of CD161 was found on CD4+ effector T cells and on CD8+ T cells but not on T regulatory cells, and experimental inactivation of the *KLRB1 gene* confirmed the enhancement of the antitumor activity of tumor-invading T cells [22]. Another important receptor is NKG2D (“natural killer group 2D”), which is downregulated in glioblastoma patients on NK cells and CD8+ T cells. This downregulation is directly mediated by increased TGF-ß expression at the transcriptional level. After tumor resection, NKG2D expression on NK cells and CD8+ T cells in the patient’s serum increased again, and postoperative increased cytotoxic activity of NK cells was confirmed in vitro [23].

In recent years, the increasing knowledge of clinically relevant immunological biomarkers has opened the possibility to distinguish between five different hypofunctional states of CD8+ T cells in tumors, namely, T cell senescence, anergy, exhaustion, tolerance and ignorance. This categorization is according to the molecular basis of dysfunction, and all hypofunctional states have been described in gliomas, especially in high-grade gliomas such as glioblastomas [2,24,25]. T cell senescence results from the continuous shortening of telomeres due to continuous T cell activation in tumors along with DNA damage such as exposure to reactive oxygen species (ROS). Since T cells lack telomerase activity, they are likely predisposed to more rapid telomere shortening and a senescent state in the tumor environment. Typical markers of T cell senescence include expression of CD57 as an indicator of terminal differentiation of T cells and downregulation of the co-stimulatory markers CD27 and CD28. A direct prognostic impact of this phenomenon has been confirmed for glioblastoma patients, as overall survival was significantly shorter in patients with a higher proportion of T cells with high CD57 and low CD28 expression [26]. T cell anergy is a collective term for various molecular cascades that lead to the common mechanism by which lymphocytes become permanently inactive after antigen contact. An important common factor in T cell anergy is the lack of significant expression of co-stimulatory ligands such as CD28, CD80 and CD86 during antigen contact, which has been found in gliomas and in various *mouse* tumor models [1,27]. One molecular cascade is the impaired regulation of the RAS/MAPK pathway in combination with the lack of expression of co-stimulatory ligands. Since this cascade was first described in T cell clones in vitro, it is referred to as “clonal anergy” [28]. Another molecular cascade, termed “adaptive tolerance”, is caused by low antigen exposure combined with low activity of the kinase Zap70 (“linker of activated T cells and phospholipase Cγ1”) and impaired calcium-induced NF_K_B signaling (“nuclear factor kappa-light-chain enhancer of activated B-cells signaling”) [1,28]. Remarkably, lower Zap70 activity has also been described in the phenomenon of T cell exhaustion, and the question has been raised whether the terms “clonal anergy” and “adaptive tolerance” should be subsumed under the term “exhaustion” [29]. T cell exhaustion is a hyporesponsive state resulting from repeated antigen exposure under suboptimal conditions. It was discovered in the context of chronic viral infection in CD8+ T cells. The two most important transcription factors involved are NFAT (“nuclear factor of activated T-cells”) and AP-1 (“activator protein 1”), as only their coupling allows for physiological differentiation of effector T cells. In the absence of NFAT/AP-1 coupling, NFAT binds to regulatory regions and induces the transcription of genes associated with a depleted state of T cells. This in turn leads to the expression of inhibitory checkpoint receptors on the T cell surface such as TIM-3 (“T-cell immunoglobulin and mucin-domain containing-3”) and LAG-3 (“lymphocyte-activation 3”), resulting in hypofunctional T cells. Hypoxia and nutrient deprivation in tumor tissue have been shown to contribute to the development of this phenomenon [1,25,29,30]. T cell tolerance under physiological conditions is a mechanism that protects immune cells through programmed induction of T cell insensitivity from aberrant autoimmunity. In gliomas, T cell tolerance can result from peripheral deletion of T cells through Fas-L-mediated apoptosis or through the immunosuppressive effect of Tregs, which in turn is induced by upregulated expression of IDO1 and the signal transducer and activator of transcription 3 (STAT3) [1,2,25]. T cell ignorance is a hypofunctional state characterized by fully functional T cells but a lack of immune response due to insufficient antigen expression or anatomic barriers. In glioblastomas, T cell ignorance has been described in cases with marked peripheral lymphopenia and in cases with sequestration of T cells [1,2,25,29,31]. All of these pathological states of T cells can occur in gliomas, although the research of their etiology and their exact interrelationship within the tumor environment of gliomas is still ongoing.

B cells are less common in brain tumors than T cells or macrophages, but in recent years, their role in gliomas has been increasingly investigated. In human and *mouse* glioblastomas, B cells are characterized by predominantly immunosuppressive activity due to overexpression of the inhibitory molecules PD-L1 and CD155 and the immunosuppressive cytokines TGF-ß and Il-10, which are known to inhibit the effector function of T cells [32,33]. In addition, there is evidence that GBM-associated myeloid-derived suppressor cells (MDSCs) promote regulatory B cell function by delivering microvesicles that transport membrane-bound PD-L1 which is taken up by tumoral B cells. This transfer of functional PD-L1 confirms the potential for B cells to suppress CD8+ T cell activation. Experimental application of anti-B cell immunotherapy with an anti-CD20 antibody resulted in prolonged survival of animals, and this finding is interpreted as evidence for a significant role of B cells in glioma progression [33]. A dual role of B cells in various tumors has been addressed, as they may exhibit pro-tumorigenic activity due to immunosuppression and impairment of cytotoxic T cell function, but impairment of T cell function by the use of anti-B cell antibodies has also been observed [34]. Of particular interest for understanding the role of B cells in tumors are tertiary lymphoid structures (TLS), which have been described in many tumors and even in gliomas [34,35]. Experimental immunostimulatory application of CD40 antibodies induced a pronounced formation of TLS with B cells as the dominant cellular component. Moreover, systemic administration of CD40 led to the accumulation of immunosuppressive CD11b+ B cells in the tumor environment, resulting in impaired CD8+ T cell cytotoxicity [35]. The opposite effect was observed in a subset of B cells expressing the tumor necrosis factor CD137 (syn. 4-1BBL). This subpopulation of B cells was able to induce antitumor cytotoxicity of CD8+ T cells, and based on this observation, an experimental B cell vaccine consisting of CD137+ B cells stimulated by CD40 and IFN-γ was developed. When combined with a blockade of PD-L1, application of this vaccine resulted in tumor eradication in 80% of treated *mice*, with significant activation of cytotoxic CD8+ T cells [36]. These results confirm that B cells in gliomas can promote immunosuppression with pro-tumorigenic effects but can also exhibit antitumorigenic activity that depends on their immunological subtypes such as CD137+ as well as various co-stimulatory treatment conditions such as an additional blockade of PD-L1 [34,35,36]. Further evidence for a significant role of B cells in glioma progression is provided by expression analysis of B cell-associated genes from a whole genome dataset of 782 high-grade gliomas, which revealed a strong correlation of the expression of five genes with patient prognosis. A clear impact on prognosis was demonstrated for the gene of the “Fc Fragment of IgG—Low Affinity II—Receptor for CD32B” (*FCGR2B gene*), which is highly expressed in the high-risk group of patients with shorter overall survival. The protein it encodes, CD32B, is a surface receptor protein on B cells known to inhibit antigen presentation and antibody production in B cells. Therefore, the authors hypothesize that this protein may be a target for immune checkpoint inhibition [37]. In summary, the current findings on lymphocytes demonstrate their central role in the development of an immunosuppressive tumor environment in gliomas. The pathophysiological endpoint is essentially the inhibition of CD8+ effector cells and NK cells (Table 1) which can occur through different signaling pathways, such as the inhibitory influence of Tregs and the majority of B lymphocytes.

## 3. Microglia and Myeloid-Derived Cells

**Figure 2:**
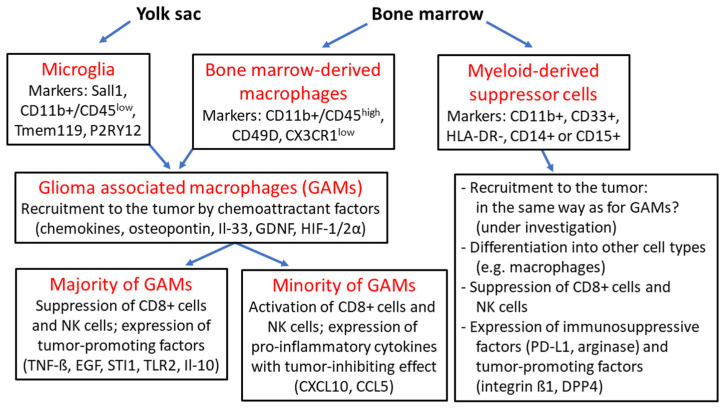
Overview of glioma-associated macrophages and myeloid-derived suppressor cells: microglia originate from the embryonic yolk sac and together with bone marrow-derived macrophages are termed “glioma-associated macrophages” (GAMs), which are recruited to the tumor by various chemoattractant factors such as chemokines, osteopontin, interleukin 33 (Il-33), glial-cell derived neurotrophic factor (GDNF) and hypoxia-inducible factors 1α and 2α (HIF-1/2α); the majority of GAMs provide an immunosuppressive tumor environment as a result of the expression of tumor-promoting factors such as tumor necrosis factor ß (TNF-ß), epidermal growth factor (EGF), stress-inducible protein 1 (STI1), Toll-like receptor 2 (TLR2) and interleukin 10 (Il-10) with suppression CD8+ cells and natural killer (NK) cells; a minority of GAMs express tumor-inhibitory cytokines such as CXCL10, CCL5; myeloid-derived suppressor cells (MDSCs) arise from hematopoietic stem cells and can differentiate into other cell types such as macrophages; MDSCs exhibit pronounced immunosuppressive activity based on the suppression of CD8+ cells and NK cells, and the expression of the checkpoint protein programmed death receptor ligand 1 (PD-L1) and arginase, as well as tumor-promoting factors such as integrin ß1 and dipeptidyl peptidase 4 (DPP4) [38,39,40,45,46,47,48,49,63,64,65,66].

Microglia are the resident macrophages in the healthy human brain. They develop from the embryonic yolk sac, after which immature amoeboid microglia migrate to the leptomeninges and central nervous system between gestational week 4 and 24. Mature microglia are characterized by longevity and have a limited capacity for self-renewal. Under physiological conditions, they perform various tasks such as functional support of neurons, phagocytosis of apoptotic cells, and immune surveillance. Under pathological conditions with impairment of the blood–brain barrier (BBB), monocytes derived from hematopoietic stem cells in the bone marrow can invade the CNS and differentiate into macrophages of myeloid origin [38,39]. In gliomas, monocyte-derived macrophages (tumor-associated macrophages, TAMs) and microglia are collectively referred to as glioma-associated microglia/macrophages (GAMs), which may account for 15–50% of all cellular components of the tumor microenvironment (TEM) according to various literature reports [2,39]. In the past, both cell types of GAMs were identified by the markers CD68, CD163 and CD200, but the frequently described method for their differentiation through detection of the markers CD11b+/CD45^low^ for microglia and CD11b+/CD45^high^ for TAMs is no longer considered sufficient for accurate confirmation of their phenotype. Other markers for microglia such as Tmem119, P2RY12 and Sall1 and also markers for monocyte-derived macrophages such as CD49D and CX3CR1^low^ have been detected (Figure 2) [2,40]. Another indication for the different distribution and abundance of microglia in gliomas is the fact that microglia are more likely to be found in peripheral tumor areas, whereas TAMs are more often found in perivascular regions and near necrotic areas in the tumor center. In experimental *mouse* models, microglia were more abundant during early glioma development, but during tumor progression, the number of CD45+ TAMs steadily increased [41,42]. Several factors known to be involved in the recruitment and chemoattraction of GAMs have been described, and to date, all of these factors appear to promote the recruitment of microglia and TAMs equally, whereas factors involved in the attraction of a single cell type are as of yet poorly understood. Important chemoattractant factors include chemokines such as CCL2, CXCL12, CX3CL1, osteopontin and its receptor integrin αvß5, interleukin-33, glial-cell derived neurotrophic factor (GDNF) and macrophage colony-stimulating factor (M-CSF or CSF1) [38,39,43,44,45,46]. Hypoxia is also an important factor in the migration of TAMs, and the hypoxic conditions of TEM are created by the expression of VEGF in combination with the hypoxia-inducible factors 1α and 2 α (HIF-1α; HIF-2α) [2,47]. TGF-ß is predominantly released by microglia and promotes glioma cell migration, as well as the release of metalloproteinase 2 (MMP2) which contributes to the degradation of the extracellular matrix and thus supports glioma invasion. Other factors that promote glioma progression and invasiveness include epidermal growth factor (EGF) expression, stress-inducible protein 1 (STI1) expression and Toll-like receptor 2 (TLR2) upregulation [39,43,48,49]. Among tumor-associated macrophages (TAMs), different types have been defined based on their molecular signature in vitro, with the M1 type representing the pro-inflammatory phenotype acquired after stimulation with Toll-like receptor 4 (TLR4) and interferon-γ (IFN-γ). The M2 immunosuppressive and pro-tumorigenic phenotype occurs after stimulation with interleukins Il-10, Il-13 and Il-14 and colony-stimulating factor M-CSF1. Further subclassification of M2 macrophages based on their expression behavior has been achieved in vitro, but there is a general consensus that these in vitro results reflect in vivo conditions only to a limited extent [2,39,40,50]. There is also a consensus that the vast majority of TAMs have tumor-supportive properties in the tumor environment, although a minority of TAMs may also have pro-inflammatory and antitumor properties [40]. Important factors expressed by TAMs in gliomas include TNF-ß, high levels of interleukins 6 and 10 (Il-6, Il-10), MMP2 and MMP9, and VEGF and VEGA, which collectively promote glioma proliferation and invasion, angiogenesis and suppression of T effector cell and NK cell activity [2,38,40,51,52]. Other effects of the expression pattern of TAMs in gliomas include promotion of the invasiveness of CD133+-positive glioma stem cells, and even upregulation of the activity of glycolytic factors such as lactate dehydrogenase A (LDHA) and glucose-6-phosphatase dehydrogenase (G6PD), leading to lactate accumulation. Furthermore, TAMs promote resistance to temozolomide therapy due to activation of the STAT3-MYC pathway through increased expression of interleukin 11 (Il-11) [51,53,54].

Research on GAMs in gliomas has uncovered a variety of new aspects over the last three to four years, each providing further insights into the molecular basis of the interplay between immune cells and the biological behavior of tumors. An important tool in this context is single-cell transcriptome analysis, which offers the possibility to analyze single cells that have been clearly identified as immune cells in a precisely localized small tissue sample. An important observation is the description of a subset of TAMs with CD169+ expression in *human* and *mouse* gliomas that produce pro-inflammatory cytokines such as CXCL10 and CCL5, leading to the accumulation of T cells and NK cells. Experimental depletion of these CD169+ TAMs resulted in shorter animal survival, confirming the antitumor role of this subset of TAMs. Similarly, Kim and colleagues found that IFN-γ expressed by NK cells was critical for the accumulation of CD169+ TAMs in glioma tissue [55]. Expression of the purinergic receptor P2RY12 has been described in several tumor types, but its significance is poorly understood. In gliomas, it is expressed in microglia but also in platelets. Different results have been obtained, with some studies supporting the view that this receptor plays a pro-tumorigenic role. In adenocarcinomas of the lung, Fan and colleagues found a significant correlation between the expression of the purinergic receptor P2Y12 (P2RY12) and the degree of tumor infiltration with immunosuppressive TAMs [56]. Expression of P2RY12 by microglia and platelets in gliomas is thought to promote the proliferative capacity and chemotaxis of tumor cells [57]. Another view of microglia in gliomas is supported by a histomorphological study of P2RY12-immunostained microglia in human glioblastomas. A survival analysis revealed significantly longer overall survival for patients with a high proportion of P2RY12-positive microglia, but this result was not significant considering the number of microglia labeled with the microglia marker Tmem119 [58]. Although the specificity of this purinergic receptor molecule for microglia and for platelets can be considered certain, its role in the pathobiology of gliomas needs further investigation [56,57,58]. DEAD/DEAH-box helicase 9 (DHX9) belongs to the family of DEAD-box proteins, which are highly conserved regulators of RNA metabolism in prokaryotes and eukaryotes. DHX9 is involved in the degradation of abnormal nucleotides to ensure DNA replication. However, under pathological conditions, DHX9 may also be involved in tumor cell survival in various tumor types. In human gliomas, DHX9 expression has been shown to increase and promote tumor cell proliferation, migration and invasion. In addition, DHX9 facilitates the infiltration of macrophages into tumor tissues and their polarization to the immunosuppressive M2 type by upregulating colony-stimulating factor 1 (CSF1). In vitro, suppression of DHX9 expression led to the downregulation of CSF1, suggesting that DHX9 should be considered as another potential target for immunotherapy, which is related to the known tumor suppressive effect of the downregulation of CSF1 [59]. Another novel aspect is the immunological significance of the most common inactivated tumor suppressor in primary gliomas, phosphatase and tensin homolog (PTEN), as high expression of PTEN showed a significant correlation with overall patient survival and even a negative correlation with the number of macrophages in the tumor. Tumor cases that contained large numbers of macrophages were more likely to be PTEN-mutated, and in vitro analyses confirmed that there was greater infiltration with immunosuppressive macrophages in cases with a loss of PTEN wild-type status. The authors highlight the prospect that PTEN status may play a key role in predicting which patients will respond to promising immunotherapies [60]. The promotion of infiltration with immunosuppressive macrophages in gliomas was also confirmed for hyaluronic acid (HA). In vitro analyses showed suppression of interleukin-1, enhancement of TNF-ß expression and enhancement of PD-L1 expression by tumors containing varying amounts of HA compared to a control group. Tumors with a large amount of HA also exhibited a greater number of immunosuppressive macrophages [61]. Of particular note are the so-called “double-positive” TAMs in glioblastomas which represent a subset of TAMs with simultaneous expression of macrophage and tumor cell markers. In vitro experiments confirmed that these double-positive TAMs are formed via phagocytosis of glioma cells by bone marrow-derived macrophages. After phagocytosis, double-positive TAMs show an immunosuppressive phenotype and transform into the M2 phenotype of TAMs, with expression of immune checkpoint proteins such as CD276 and PD-L1. Although double-positive macrophages with simultaneous expression of tumor cell and macrophage markers have been described in various tumor types, this phenomenon has not been explored to any significant extent in gliomas. Wu and coworkers consider the role of phagocytosis of tumor cells by macrophages as an important phenomenon driving TAM-mediated immunosuppression in glioblastomas (Table 2) [62].

Myeloid-derived suppressor cells (MDSCs) are a population of immature myeloid cells that arise from hematopoietic stem cells or other myeloid progenitor cells and can differentiate into mature cells such as granulocytes, macrophages and dendritic cells. MDSCs represent a heterogeneous group of cells whose common feature is the expression of CD11b+ and CD33+ and the absence of expression of the human leukocyte antigen—DR isotype (HLA-DR). The two major subsets are granulocytic G-MDSCs expressing CD15+ and monocytic M-MDSCs expressing CD14+, although a total of six molecular subtypes of human MDSCs have now been described based on their distinct expression patterns [63,64,65]. MDSCs have the ability to suppress the cytotoxic activities of natural killer (NK) cells and the immune response mediated by CD4+ and CD8+ T cells, and they can even induce apoptosis of T cells [65]. MDSCs play a central role in tumor-induced immunosuppression in various cancers, and especially in glioblastomas, considerable infiltration with a high frequency of G-MDSCs and M-MDSCs has been reported in tumor tissue and peripheral blood of patients [2,65,66]. Programmed cell death ligand 1 (PD-L1) is upregulated on MDSCs in glioblastoma tumor tissue, and co-culture experiments have confirmed that these MDSCs can induce PD-1 expression on T cells [66]. The number of MDSCs in glioblastoma tissue and peripheral blood inversely correlates with overall survival and patient prognosis [67]. Although there is limited literature on the infiltration of MDSCs in different glioma types and tumor grades, there is no doubt that the extent of MDSC infiltration of tumors generally correlates with clinical cancer stage, histological tumor grade and patient prognosis [2,65]. MDSCs use different mechanisms to dampen antitumor immunity and promote glioma progression. In addition to participating in immune checkpoint blockade through increased expression of PD-L1 [66], increased expression of arginase leads to the degradation of L-arginine in tumor tissue and peripheral blood. L-arginine is required for normal T cell function via translation of the T cell CD3 zeta chain, and degradation of L-arginine inhibits T cell activity and proliferation [63,65]. Other mechanisms include secretion of nitrite oxide and reactive oxygen species (ROS), leading to decreased T cell activity due to various effects such as the downregulation of CD44 and CD162 and impairment of interleukin L2 signaling by T cell receptors [2,63]. Other upregulated factors involved in promoting glioma progression include integrin ß1, which can form complexes with multiple α-subunits, and dipeptidyl peptidase 4 (DPP-4), a serine peptidase that can cleave various substrates such as chemokines. Although the exact mechanism of both compounds in relation to the activity of MDSCs is still unclear, their experimental inhibition resulted in the downregulation of ERK signaling and inhibition of MDSC migration to tumor cells [68]. Another negative regulator of the ERK pathway is dual-specificity phosphatase 3 (DUSP3), which is also involved in neovascularization in tumors [69]. MicroRNA miR-1246 released from exosomes in gliomas upregulates the DUSP3/ERK pathway, leading to activation of the immunosuppressive function of M-MDSCs [70]. Although high expression of exosomal miR-1246 is known to correlate with pathological tumor grade and inversely with prognosis in various cancers [71,72], the precise role of this microRNA and DUSP3 signaling in glioma biology needs further investigation. An important observation is the downregulation of the surface ectoenzyme vanin-2 (VNN2) on M-MDSCs in the peripheral blood of patients with high-grade gliomas. Vanins are a group of proteins with pantetheinase activity involved in the regulation of inflammatory processes. Vanin-2 is expressed on normal M-MDSCs and is involved in transendothelial migration of neutrophils, whereas the reasons for its downregulation in M-MDSCs from glioma patients are not clear to date. The potential role of vanin-2 as a useful marker for tumor diagnosis and even its role as a therapeutic circuit to curb the immunosuppressive capacities of M-MDSCs in gliomas has been addressed [73]. Taken together, current findings on bone marrow-derived myeloid cells demonstrate their predominantly immunosuppressive activity within the TEM of gliomas, which is pronounced in both MDSCs and macrophages. In addition to the inhibition of effector T cells and NK cells, these cells also express numerous tumor-promoting factors such as TNF-ß or Il-10. In contrast, a comparatively small number of macrophages show expression of tumor-inhibiting factors, and to date, there are also different reports regarding tumor-promoting or tumor-inhibiting activity in microglia.

## 4. Glioma Stem Cells

Cancer stem cells represent a small cellular compartment in malignant tumors, and their properties strongly overlap with those of somatic stem cells, such as the ability to self-renew, sustained proliferative activity, expression of stem cell markers and the ability to generate progeny of different cell lineages. The ability to form tumors after experimental secondary transplantation is another important property of various cancers and has led to the “stem cell hypothesis”. This theory is based on the assumption that pluripotent and self-renewing stem cells represent the top of the cellular hierarchy in the tumor and are responsible for the continuous growth and maintenance of the tumor [74,75,76]. The stem cell hypothesis is not the only model of tumor evolution, as two other models are discussed in the literature. According to the older “stochastic model”, all tumor cells are assumed to drive tumor progression equally. The more recent “cancer stem cell evolution model” assumes that due to extrinsic or intrinsic factors such as cell environment, therapy or genomic alterations, cancer stem cells have the ability to produce new and phenotypically distinct cancer stem cell clones capable of promoting tumor progression. Importantly, these models are studied separately in different tumor types and a different scientific understanding of “cancer stem cells” has led to different terminology of this cell type, so the terms “tumor-initiating cells” or “stem cell-like cells” are also used in the literature [74,77]. In gliomas, the most commonly used stem cell marker is prominin-1 (CD133), a glycoprotein and neural stem cell marker involved in cellular differentiation and epithelial to mesenchymal transition. In glioma stem cells, this marker often shows false-negative results due to weak or absent expression of CD133 in the G0 and G1 phase [76]. Another glioma stem cell marker is A2B5, which is expressed on the surface of oligodendrocyte progenitor cells under physiological conditions. Glioma cells with A2B5+ expression are able to differentiate into cells with phenotypic similarities to neurons, astrocytes and oligodendrocytes. Human astrocytic gliomas with marked A2B5+ expression showed poorer clinical outcome in patients with higher recurrence rates [78,79]. Stage-specific embryonic antigen-1 (SSEA-1, CD15) is a neural stem cell marker and is expressed in stem cell-like cells of glioblastomas in vitro. To date, there is no report of prognostic significance of CD15 [74,76,80,81]. CD44 is a receptor for hyaluronic acid and is frequently expressed in glioma stem cells. A retrospective analysis of clinical reports of gliomas confirmed that CD44 expression was significantly predictive of shorter overall survival in patients with WHO grade II and III gliomas but not in patients with glioblastomas [82]. In contrast, the signal transducer and neural stem cell marker CD24 significantly correlates with a more favorable prognosis of glioma patients, and it has been suggested that it may be a marker that is specifically upregulated in IDH-mutated gliomas [83]. The intermediate filament protein nestin is also a neural stem cell marker expressed in many glioblastomas. Reports of significant correlation of its expression with tumor behavior and patient prognosis have yielded mixed results, although a large meta-analysis found that nestin expression significantly correlated with poorer prognosis in patients with gliomas of various tumor grades (II-IV) [78,84]. The cytosolic protein aldehyde dehydrogenase 1 (ALDH1) is a glioma stem cell marker and is involved in the metabolism of carboxylic acid and the conversion of retinol to retinoid acid. It is not a specific stem cell marker, as it is also expressed on normal astrocytes. There are several molecular subtypes of ALDH1, and in particular, the enzymatic activity of the ALDH1A3 subtype has been shown to induce the expression of tissue transglutaminase (tTG), a GTP-binding protein involved in glioma stem cell survival and chemotherapy resistance. The authors hypothesize that future studies may find a similar role for other ALDH1 family members in various cancers [85]. Low activity of the proteasome, the primary organelle for targeted protein degradation, has been observed in cancer stem cells from various tumors. In vitro studies of cancer stem cells from human colorectal cancer and from *mouse* breast cancer have shown an increased capacity for local tumor formation, especially in cells with low proteasome activity [86,87]. In patient-derived glioblastoma cells, a significantly greater ability to form tumors in *mouse* xenografts has been observed in cells with low proteasome activity [87]. Therefore, evidence of decreased proteasome activity is also considered a marker for cancer stem cells.

**Figure 3:**
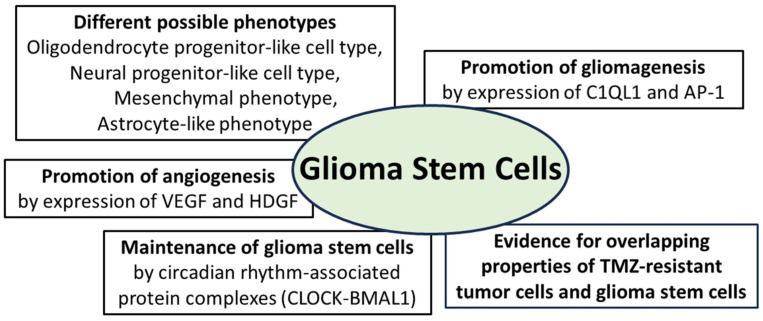
Essential properties of glioma stem cells: they can adopt different phenotypes and promote gliomagenesis through expression of the synapse-associated protein complement component 1 q subcomponent-like 1 (C1QL1) and the activator protein (AP-1); angiogenesis is promoted by expression of vascular endothelial growth factor (VEGF) and hepatocytic growth factor (HDGF); maintenance of glioma stem cells is promoted by protein complexes from the group of circadian-associated CLOCK proteins (“circadian locomotor output cycles kaput”); furthermore, there is evidence for overlapping molecular properties of TMZ-resistant tumor cells and glioma stem cells [74,75,76,77,90,94,101,102,103].

Regarding new findings on glioma stem cells in recent years, it is important to highlight the important new view that glioma stem cells do not have a constant phenotype throughout tumor progression but undergo developmental programs that lead to changes in their phenotype, including the expression of stem cell markers [74,88]. A meta-review based on single cell RNA sequencing data of glioblastomas confirmed that different phenotypic states of glioma stem cells are associated with strong expression of specific markers. The oligodendrocyte progenitor-like cell type predominated in stem cells in which CD133 had the highest expression level among all stem cell markers. In the cells in which nestin had the highest expression, an astrocyte-like phenotype was the predominant cell type. A mesenchymal cell type was predominant in stem cells that showed marked expression of CD44, whereas a neural progenitor-like cell type was evident in cells with marked expression of CD24 [89]. This phenotypic difference between stem cells exhibiting different expression patterns of stem cell markers was confirmed in two glioma models derived from *human* glioma stem cells. Whole-exome and single-cell RNA sequencing methods demonstrated that multistep transcriptional reprogramming of the stem cell population, leading to a change in tumor phenotype, drives tumor progression. The high proliferation activity of stem cells persisted throughout tumor progression. Genetic analysis revealed two novel tumor-promoting factors that the authors believe are potential key factors in the early progression of human gliomas: Complement component 1 q subcomponent-like 1 (C1QL1) is a synapse-associated protein with little understood function. It is thought to play a role in the interaction between gliomas and neurons, while activator protein-1 (AP-1) is thought to play a central role in switching from neurogenic to gliogenic cells [90]. Further investigation of the spatial distribution of glioma stem cell phenotypes in human malignant gliomas has shown that the tumor core is enriched in oligodendrocyte progenitor-like cells, whereas radial glial stem cells are enriched in the invasive tumor region, which still contains many neuronal cells. Transcriptome analyses revealed that the protein kinase “family with sequence similarity 20, member C” (FAM20C) is an important factor mediating the invasive growth of these glial stem cells in the invasive neuron-rich tumor region. The authors consider these results as a contribution to the understanding of spatial tumor architecture in relation to stem cells in malignant gliomas [91]. Another contribution in this context is the detection of glycerol-3-phosphate dehydrogenase 1 (GPD1) in glioma stem cells but not in normal neural stem cells from an experimental glioma model. GPD1 has been detected in tumor cells of various tumor types and is involved in the generation of glycerol within an active glycolysis pathway that is a hallmark of neoplastic cells. In the glioma model, cells expressing GPD1+ were predominantly found at the tumor margin of newly developing tumors, but their numbers decreased significantly in fully developed tumors. Therefore, these tumor cells are considered as quiescent glioma stem cells which participate in tumor initiation as GPD1+ expressing cells at the tumor margin but transition to a quiescent state with a loss of GPD1+ expression in later tumor stages [92]. The problem of chemoresistance of gliomas to temozolomide (TMZ) has been addressed in the past, including discussions of the possible mechanisms of drug resistance in the context of glioma stem cells [93]. In a recent study of temozolomide-resistant glioma cell colonies derived from a TMZ-exposed glioma cell line, the expression of stem cell markers such as CD133 and nestin was significantly higher in the newly formed tumor cells compared to their parent cells prior to TMZ exposure. Within the newly formed tumor cell colonies, the majority of cells expressed stem cell markers and showed TMZ resistance, but a minority of tumor cells expressing stem cell markers were chemosensitive. The authors consider these observations as evidence for the partially overlapping properties of glioma stem cells and TMZ-chemoresistant glioma tumor cells, opening a new perspective for the use of appropriate cell subpopulations as therapeutic targets for gliomas (Figure 3). The need for further investigation into the biological nature of the present subpopulations with and without resistance to therapy is specifically addressed [94].

The interaction of glioma cells with the tumor vasculature is well known, as their release of regulatory growth factors such as vascular endothelial growth factor (VEGF) or hepatocytic growth factor (HDGF) promotes angiogenesis. A subset of stem cell-like glioma cells with CD133+ expression shows an additional expression of vascular endothelial cadherin (CD144). These cells have the phenotype of endothelial progenitor cells and are capable of maturing into endothelial cells [95,96,97]. Although this phenomenon has been known for several years, the exact molecular mechanism is still largely unknown. Wingless-related integration site family member 5A (WNT5A) has been shown to be involved in endothelial differentiation during embryogenesis but also in the transition of glioma stem cells to endothelial cells. Experimental knockdown of WNT5A resulted in the suppression of angiogenesis and tumor progression and decreased the stimulation of glioma stem cells to differentiate into endothelial cells. Regarding the molecular cascade, the frizzled-4 receptor mediates the effect of WNT5A on endothelial differentiation and angiogenesis via the GSK3β/β-catenin/epithelial-mesenchymal transition signaling pathway [98]. Recently, the influence of transcription factors associated with circadian rhythm on the maintenance of glioma stem cells, as well as tumor stem cells of other tumor types such as acute leukemia, has been demonstrated. The importance of these genes, belonging to the group of “*CLOCK genes*”, has been confirmed by their downregulation leading to cell cycle arrest and apoptosis of tumor stem cells [99,100]. CLOCKs (“circadian locomotor output cycles kaput”) represent a group of transcription factors that are able to form complexes with each other to support the circadian rhythm phenomenon (Figure 3). The four main groups of circadian transcription factors are CLOCK, BMAL1, PER and CRY, and some of these factors or protein complexes activate transcription (CLOCK-BMAL1 complex) or even inhibit it (PER; CRY) [101,102]. Further insights into molecular signaling have been gained as the CLOCK-BMAL1 complex not only maintains glioma stem cells but also promotes microglial invasion into the tumor environment [103]. Regarding the promotion of angiogenesis in malignant gliomas, the CLOCK-BMAL1 complex was found to lead to transcriptional upregulation of periostin (POSTN), which promotes angiogenesis by activating TANK-binding kinase 1 (TBK1) in endothelial cells. An experimental blockade of the CLOCK-BMAL1 complex inhibited tumor progression and angiogenesis. The authors interpret this result as confirmation that the CLOCK-POSTN-TBK1 axis is involved in tumor angiogenesis and point to the need for further investigation into the precise molecular interaction between CLOCK, POSTN and TBK1 [101]. The summarized findings on glioma stem cells demonstrate the expression of tumor-promoting and angiogenesis-promoting factors. The ability to change their phenotype as well as the influence of circadian-regulated protein complexes on their maintenance are also among the key current scientific findings.

## 5. Other Cellular Components of the Tumor Environment in Gliomas

In addition to lymphocytes, microglia, myeloid cells and glioma stem cells, there are other cell types that have been shown to communicate with the tumor at the molecular level. These cell types include neutrophils and mast cells, cancer-associated fibroblasts and dendritic cells, as well as neurons, astrocytes and oligodendrocytes, which represent the non-neoplastic cell types of the CNS and whose interactions with glioma tumor tissue are increasingly being investigated. Neutrophils are frequently found in the central tumor regions of gliomas, and a high abundance of neutrophils significantly correlates with tumor grade and with poorer prognosis of patients [104,105]. Two subtypes of tumor-associated neutrophils have been proposed: antitumor N1 cells induced by IFN-ß and tumor-promoting N2 cells induced by TGF-ß. To date, however, it is unclear whether this polarization actually represents two distinct subsets of tumor-associated neutrophils or whether it is due to the regulation of different cytokines within the TEM [106,107]. Currently, the prevailing view is that a marked presence of neutrophils in malignant tumors is associated with a poorer prognosis. Moreover, in vitro and in vivo studies have confirmed that expression of the S100A4 protein by neutrophils promotes the progression of gliomas, leading to a transition to a mesenchymal glioblastoma phenotype and an increased proliferation rate. Experimental downregulation of S100A4 expression in vitro and in vivo resulted in blockage of neutrophil-promoted tumor progression. S100A4 is involved in several physiological processes, including cell survival, motility and differentiation. An association between S100A4 expression and tumor aggressiveness has been confirmed for several tumor types, but the exact mechanism of mesenchymal transition in gliomas promoted by S100A4 protein is unclear. An interaction with metalloproteinase 9 (MMP9) is discussed in [105]. A significant association of TERT mutations in gliomas with a higher proportion of tumor-infiltrating neutrophils has also been confirmed. In particular, compared with tumors without TERT mutations, IDH wild-type glioblastomas with TERT mutations have a greater number of neutrophils with marked expression of the cytokines CXCR2, CXCR4, CCL2, CCL5 and MMP9. Of note, the number of neutrophils was not increased in the peripheral blood of patients with TERT-mutated gliomas, suggesting that tumor infiltration by neutrophils is due to cytokine chemotaxis. These data are interpreted as a correlation of TERT mutation status with immune response and with the infiltration of neutrophils in the microenvironment of IDH wild-type glioma, but further exploration of the underlying molecular mechanism is required [106,108]. Like tumor-associated neutrophils, mast cells express the cytokine CXCR4, and they are present more largely in high-grade gliomas than in low-grade gliomas. Since CXCR4 is the corresponding receptor for the cytokine CXCL12 expressed by glioma tumor cells, it is suggested that mast cells may be attracted to the tumor via the CXCL12/CXCR4 axis [109]. Plasminogen activator inhibitor 1 (PAI-1) is involved in the regulation of the plasminogen–plasmin system and in setting the locomotion and migration direction of cells. It is able to form a complex together with proteins of the fibrinolytic system and low-density lipoprotein receptor-related protein 1 (LRP1). In human gliomas, a positive correlation has been found between the number of mast cells and PAI-1 concentration, and tumor infiltration by mast cells could be attenuated by neutralizing PAI-1. Furthermore, activation of the interaction between PAI-1 and LRP1 leads to increased phosphorylation of STAT3 and exocytosis in mast cells. It is suggested that degranulation of mast cells initiated via the PAI-1/LRP1/STAT3 axis leads to release of additional chemokines that trigger tumor infiltration of other immune cells and tumor progression [110]. Serglycin is an intracellular proteoglycan localized in the granules of mast cells and secreted by their degranulation. High serglycin expression in glioblastomas inversely correlates with patient prognosis. In other tumor types, serglycin has been confirmed to promote metastatic tumor growth, and in vitro observations of human glioma tumor cells have shown increased release of the glioma stem cell marker CD44 mediated by serglycin. According to the authors, these observations suggest that serglycin released by mast cells may support and further promote glioma cell progression and spread [111].

Like neutrophils and mast cells, cancer-associated fibroblasts (CAF) are also a component of the tumor environment (TEM) in various cancers. The term “cancer-associated fibroblasts” encompasses all fibroblasts detected in TEM, which may be resident tissue fibroblasts or transdifferentiated fibroblasts derived from non-fibroblastic cell lines such as endothelial cells. Typical marker proteins include smooth muscle actin α (α-SMA), fibroblast activation protein (FAP) and platelet-derived growth factor receptor (PDGFR), whereas CAFs are negative for CD45 [112]. In contrast to CAFs in peripheral tumors, fibroblasts were thought to be absent in gliomas. However, a recent study of 16 human glioblastomas confirmed the presence of CAFs in the tumors using single-cell RNA analysis and spatial transcriptomics. CAFs were enriched in perivascular regions of the tumors and exhibited proximity to glioma stem cells, endothelial cells, and M2 macrophages. Secretome analysis of CAFs and additional in vitro analyses confirmed that CAFs are chemotactically attracted to glioma stem cells via PDGFR and TGF-ß signaling pathways and that CAFs exhibit pro-tumoral effects, including the upregulation of HIF-α signaling and promotion of glioma stem cell proliferation. Shorter overall survival of newly diagnosed glioblastoma patients was confirmed for those patients who had high intratumoral expression of actin alpha 2 (ACTA2), which was one of the highly expressed CAF markers in this study [113]. Two large statistical transcriptome and genomic analyses based on large databases of human gliomas revealed additional CAP markers whose expression correlates with patient prognosis. In particular, two clusters were observed in low-grade gliomas, with the first cluster representing cases with a poorer prognosis, a greater number of CAFs and marked expression of chitinase-3-like protein 1 (CHI3L1), a glycoprotein known to be associated with activation of antiapoptotic signaling pathways [114]. Statistical analysis of another database containing both low-grade and high-grade gliomas confirmed higher expression of a large number of CAF-associated cytokines in the high-grade group [115]. Runt-related transcription factor 1 (RUNX1) is a member of the RUNX family of transcription factors involved in various functions such as skeletal development or immune cell maturation. Overexpression of RUNX1 is seen in many cancers, and the extent of expression is significantly related to the extent of CAF infiltration in tumor tissue. In gliomas and even colorectal carcinomas, RUNX1 expression inversely correlates with prognosis. However, of the 33 different cancer types studied, many tumor types such as breast and lung cancer showed a positive correlation between the level of RUNX1 expression and prognosis. The need for further tissue studies to clarify the molecular mechanisms in the interaction between RUNX1 and CAFs is highlighted separately for different cancer types [116].

Dendritic cells (DCs) are antigen-presenting cells that act as messengers for extracellular antigens. Using cross-presentation, DCs transmit extracellular antigens via MHC class 1 complexes to CD8+ cytotoxic T lymphocytes, leading to their activation. Several types of DCs are known, derived from DC progenitor cells, from plasma cells or from monocytes. In the CNS, dendritic cells are found in vascular regions such as the choroid plexus and meninges but not in normal brain parenchyma. In many pathological conditions such as neurodegenerative diseases or brain tumors, DCs occur within brain lesions. The exact route by which they enter the brain is not yet known, but a pathway through endothelial venules or an afferent lymphatic system has been postulated [104,117]. Regarding the investigation of the specific role of DCs in gliomas, an important breakthrough seems to be the recent finding of the role of fibrinogen-like protein 2 (FGL2) expressed in immune cells and in glioma cells, and this expression correlates with a worse clinical outcome. Expression of FGL2 by glioma cells leads to the suppression of the dendritic cell marker CD103 by blocking granulocyte macrophage colony-stimulating factor (GM-CSF), which in turn leads to defective differentiation of DCs. In vitro, downregulation of FGL2 in tumor cells did not affect tumor cell proliferation, but in an experimental glioblastoma *mouse* model with evidence of DCs with defective differentiation, downregulation of FGL2 impaired tumor progression. The authors concluded that FGL2 serves as an onco-immune target and that its overexpression in glioma cells affects the differentiation of DCs [118]. In human gliomas with IDH mutations and IDH wild-type, DCs were investigated by transcriptomic and proteomic profiling, by single cell sequencing and by flow cytometric analyses. In addition, DC functionality was investigated in an experimental *mouse* model. The results showed that DCs in IDH-mutated gliomas exhibit an immature cellular state with reduced expression of the antigen presentation protein-signature, whereas high expression was observed in IDH wild-type gliomas. Expression of R-2-hydroxyglutarate impaired the differentiation and function of DCs in IDH-mutated gliomas and specifically suppressed MHC class I/II-mediated antigen cross-presentation and co-stimulation by IL-6. The authors consider these dysfunctional DCs as a potential therapeutic target for immunotherapy in IDH-mutated gliomas [119]. Thyroid-stimulating hormone (TSH) accelerates genomic instability and angiogenesis in thyroid cancer by binding to the TSH receptor (TSHR), and a similar role of TSH is expected in other cancers. The TSHR is expressed in various organs and tissues outside the thyroid gland, such as the brain, bone marrow and bone. Single-cell transcriptome and ELISA analyses of DCs in the tumor environment of thyroid cancer, breast cancer, melanoma and glioma showed high expression of TSHα and TSHß2, and these DCs were the major source of TSH in the tumor environment of all tumors studied. TSH released by DCs promoted proliferation, especially in thyroid tumors and gliomas, which are tumors with high TSHR expression. Moreover, TSH induced PD-L1 expression via the TSHR-JUN pathway and promoted the immunosuppressive environment of tumors, whereas TSHR inhibitors reversed tumor progression. Wu and coworkers address the prospect of TSHR as a target for cancer immunotherapy [120].

**Figure 4:**
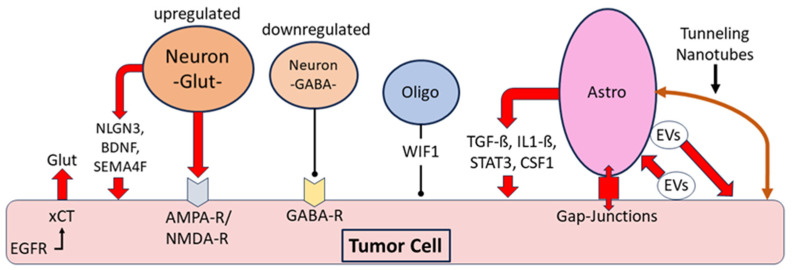
Interaction between tumor cells and non-neoplastic neurons, oligodendroglia and astrocytes in gliomas: Release of glutamate (Glut) by neurons promoting glioma growth, and additional glutamate release by glioma cells via the cystine glutamate antiporter xCT and its interaction with epidermal growth factor receptor (EGFR). Downregulation of the γ-aminobutyric acid system, particularly parvalbumin-containing “fast-spiking” GABAergic interneurons; promotion of tumor growth by neuronal release of the mitogens neuroligin3 (NLGN3), brain-derived neurotrophic factor (BDNF) and the transmembrane protein semaphorin-4F (SEMA4F). Evidence for inhibition of tumor proliferation by Wnt inhibitory factor 1 (WIF1) expressed by non-neoplastic oligodendrocytes. Astrocytic release of the pro-tumorigenic cytokines transforming growth factor ß (TGF-ß), interleukin 1-ß (Il1-ß), signal transducer and activator of transcription 3 (STAT3), and colony-stimulating factor 1 (CSF1). Intercellular communication of astrocytes with tumor cells via gap junctions, bidirectional exchange of extracellular vesicles (EVs) and tunneling nanotubes [121,122,123,124,125,126,134,135,136,137,138,139,140,141,142,143].

Non-neoplastic neuronal cells can exert a tumor-promoting effect on gliomas by releasing the excitatory neurotransmitter glutamate. Moreover, the release of glutamate from glioma cells via the cystine glutamate antiporter xCT increases the glutamate concentration in glioma tissue. The expression of xCT is upregulated by an interaction with the epidermal growth factor receptor (EGFR) (Figure 4) [121,122]. The promotion of glioma growth and invasiveness is supported by glutamate activation of the glutamate receptors AMPA and NMDA on glioma cells, and expression of both receptors is highest at the infiltrating edge of tumors [122]. Three major neuronal mitogens that contribute to the promotion of glioma progression have been studied: neuronal surface protein neuroligin3 (NLGN3), brain-derived neurotrophic factor (BDNF) and transmembrane protein semaphorin-4F (SEMA4F). These proteins upregulate the molecular signaling pathways of gliomas such as the mTOR pathway as well as glutamatergic signaling pathways (Figure 4) [123,124]. In addition to aberrant glutamatergic signaling in the glioma microenvironment, γ-aminobutyric acid (GABAergic) signaling is dysregulated. Tumor cells in malignant gliomas contain GABA receptors, and endogenous GABA released from tumor cells can attenuate tumor proliferation. However, in vitro studies confirmed a dysfunctional influence of glioma cells on GABAergic neurons due to a hyperactivated mTOR signaling pathway, leading to decreased electrophysiological activity, particularly of parvalbumin-containing “fast-spiking” GABAergic interneurons (FS). Experimental optogenetic stimulation of FS neurons in a glioma *mouse* model confirmed this dysregulation of the GABAergic system in gliomas [125,126]. Decreased expression of potassium chloride cotransporter 2 (KCC2) is the major reason for the inhibition of GABAergic neurons, and this decreased KCC2 expression leads to increased intraneuronal chloride concentration and attenuation of the inhibitory effect of GABA signaling. Downregulation of KCC2 was induced by increased activity of glutamatergic neurons with upregulated NMDA receptors and calcium influx [122,127]. These observations confirm complex changes in the interplay between excitatory and inhibitory non-neoplastic neurons in the tumor environment, leading to the promotion of tumor progression. The Na^+^/Ca^2+^ exchanger (NCX) is an antiporter system located in the cell membrane of neurons, glioma cells and other cell types [128,129]. There is evidence that NCX supports glioma growth by inducing hyperexcitability of glutamatergic neurons, by blocking apoptosis of glioma cells and by acidification of the surrounding tissue with promotion of tumor cell migration. Under physiological conditions, NCX is responsible for calcium homeostasis by transporting calcium ions out of the cell in exchange for sodium ions. In tumor cells, this ion exchange can be reversed, leading to the activation of tumor-promoting metabolic pathways. Although the exact downstream molecular cascade of NCX in tumors is still largely unclear, several studies confirmed the tumor-promoting effect of NCX on glioma cells with the induction of hyperexcitability of neurons [128,129,130,131].

Although gliomas usually arise from astrocytic or oligodendrocytic progenitor cells, little is known about the interplay between gliomas and mature non-neoplastic oligodendrocytes in the tumor environment. At the molecular level, the protein tyrosine phosphatase receptor zeta 1 (PTPRZ1) has been shown to be an important link between oligodendrocytes and gliomas, as PTPRZ1 is involved in oligodendrocyte maturation and differentiation, and upregulation of its expression is known to be a major factor in the development of various cancers. Several signaling pathways such as the ß-catenin or the mTOR pathway have been shown to be among the downstream signaling pathways activated by PTPRZ1, and this activation occurs in glioma cells as well as in the stimulation of oligodendrocyte progenitor cell growth [132]. Despite this molecular link represented by the protein PTPRZ1, there is no clear evidence in TEM of a direct interaction between mature oligodendrocytes and gliomas. For Wnt inhibitory factor 1 (WIF1), which is expressed by non-neoplastic oligodendrocytes in gliomas, it is suggested that this factor may contribute to the inhibition of proliferation and further tumor growth, but exploring the molecular link between gliomas and the expression pattern of non-neoplastic oligodendroglia remains a task for future studies [133,134]. Further insights have been gained regarding the link between gliomas and non-neoplastic astrocytes. It has been shown that reactive astrogliosis occurs very early during glioma development, starting at the tumor boundary with the expression of typical astroglial proteins such as glial fibrillary acid protein (GFAP), nestin, vimentin and the gap junction marker connexin 43. At a later stage, astroglia express tumor-promoting proteins such as TGF-ß and STAT3, as well as the immunosuppressive cytokine interleukin1-ß [135]. The activity of the voltage-gated potassium channel protein Kv1.3 is increased in gliomas, which impairs physiological glutamate buffering of astrocytes and promotes tumor cell migration [136]. Glioma progression is also supported by astrocytic release of CC chemokine ligand 2 (CCL2) and colony-stimulating factor 1 (CSF1), leading to recruitment of tumor-associated macrophages with a pro-tumor phenotype [137]. Of all of the cell types in the tumor environment in gliomas, non-neoplastic astrocytes in particular communicate significantly with tumor cells via intercellular transport using extracellular vesicles (EVs), gap junctions and nanotubes. Glioma cell-derived EVs taken up by astrocytes increase their migratory capacity and activate oncogenic signaling pathways such as epidermal growth factor (EGF) and mitogen-activated protein kinase (MAPK) [135]. An in vitro study comparing exposure of astrocytes to EVs from glioma cells and from normal epithelial cells found significant differences attributable to the increased migratory capacity of astrocytes treated with EVs from glioma cells, along with the release of several cytokines that promote tumor cell growth [138]. Connexin 43 is the major component of gap junctions, which are intercellular channels composed of a hexamer of basic proteins, connexins, which establish an intercellular connection between the cytoplasm of two adjacent cells. Glioma cells and adjacent astrocytes express connexin 43, and elevated mRNA levels of connexin 43 have been associated with the promotion of tumor invasion and unfavorable prognosis [139]. An in vitro analysis involving a co-culture of glioma cells and astrocytes showed increased resistance of glioma cells to temozolomide chemotherapy, and this effect was dependent on the presence of gap junctional communication between astrocytes and glioma cells [140]. In another in vitro analysis, a considerably fast formation of gap junctions between cocultured glioma cells and astrocytes was observed within 24 h of seeding. Cells expressing connexin 43 were predominantly located at the tumor boundary, and furthermore, inactivation of connexin 43 resulted in a marked reduction in tumor growth, suggesting that intercellular communication of glioma cells with astrocytes via gap junctions is a driving force for tumor invasion and progression [141]. Tunneling nanotubes (TNs) are another type of intercellular tubular structures formed by elongated protrusions of cell membranes that connect between two cells over a distance of up to 500 µm. TNs have been reported to occur in many pathological conditions such as HIV and neurodegenerative diseases, but little is known about their role in cancer. Co-culture systems with glioma cells and astrocytes have demonstrated a predominantly astrocytic origin of TNs and a dependence of TN formation on TP53 activity. Knockdown of TP53 significantly reduced the number of TNs, whereas knockdown of the P53 inhibitor MDM2 resulted in a significant increase in the number of TNs [142]. Regarding the role of TNs in gliomas, different observations have been made; in one study, the proliferation of glioma cells was significantly reduced by the presence of TNs [142]. In another study, glioma cells were shown to adapt surrounding astrocytes to hypoxic and metabolic tumor conditions via TNs. The authors concluded that TNs are an efficient cell-to-cell communication system used by cancer cells to adapt the microenvironment to the invasive nature of the tumor [143]. In addition, the study confirmed the transport of mitochondria via TNs. The authors hypothesize that TN-mediated transport of mitochondria alters the content and function of these organelles in the targeted non-tumor cell, but it is yet unclear whether these mitochondria are functional in the recipient cell [143]. The central finding of the cell types discussed in this section is that in addition to lymphocytes, microglia, myeloid cells and glioma stem cells, all other cell types also contribute significantly to the predominantly immunosuppressive and tumor-promoting TEM in gliomas. The presence of neutrophils, mast cells and cancer-associated fibroblasts correlates with the progression of gliomas, as does the expression of TSH by dendritic cells. The tumor-promoting influence of neurons and astrocytes must be particularly emphasized, especially the dysregulation of the transmitter system and the diverse pathways of intercellular communication between astrocytes and glioma cells.

## 6. Discussion

Much new knowledge has been gained on the topic of the tumor environment (TEM) in gliomas and the molecular pathways involving the cellular components of the TEM. The methodological spectrum of examination methods of TEM cells comprises the previously known methods of analyzing tumor cells including all histological methods, antibody-based and spectroscopic methods of proteomics and metabolomics, and flow cytometry and sequencing techniques. A comparatively new sequencing technique is nanopore sequencing which does not require a preceding polymerase chain reaction or labeling of the sample. Nanopore sequencing has occasionally been used to study gliomas, one example being the detection of mutations of the gene for the “cyclin-dependent kinase inhibitor 2A/B (CDKN2A/B) in IDH-mutated gliomas [144]. This method has yet to be applied to TEM cells. A challenge is still the comparatively high error rate in the reading of the base sequence, but this is constantly being improved by increasing the reading length of the sequences [144]. The outstanding new method for examining the cells of the TEM is the complex of single-cell analysis, which can basically be carried out in three different ways. The use of microdissection is possible, but the acquisition of a complete single cell is difficult and, especially in the case of single-cell analysis, is associated with the potential possibility of partial thermal damage to the genome and transcriptome. Flow cytometric separation of the labeled cell types enables reliable separation and specific examination of the individual cell types and is frequently used for the cells of the TEM; an example is the examination of CD169-positive macrophages, which exhibit antitumor activity [55]. A special feature is spectral flow cytometry, which is equipped with a spectrograph instead of a mirror and enables the analysis of multiple proteins in defined cell types. One example of the application to TEM cells is the analysis of dysfunctional dendritic cells in gliomas [119]. Microfluidics also represent a newer method for single-cell analysis. The common feature of all methodological approaches is the directional separation of cells or molecules within a carrier fluid at the chip level, whereby two- or even multidimensional concentration spaces can be investigated. The method has so far only been used sporadically on TEM cells. An example of such an application is the investigation of the influence of a micro-RNA (miRNA-124) on the growth of the glioblastoma cell line U373MG in vitro, whereby the micro-RNA was delivered by extracellular vesicles (EVs) via microfluidic channels to the tumor cells, which led to growth inhibition of the glioma cells [145]. Continuous methodological refinement of the various approaches has enabled largely reliable analysis of concentration gradients and the provision of pathways for the delivery of defined substances and molecules to tumor cells [145,146].

Tumor-associated macrophages (TAMs) have been the focus of scientific interest in previous years, but many recent findings have shown that the pathophysiological properties of all other cell types within the TEM should be considered equally important. This applies to lymphocytes and their subtypes and begins with new insights into the nature of their recruitment into TEM. An important experimental finding in this context is the confirmation that lymphocytes migrate from deep cervical lymph nodes into glioblastomas after stimulation with vascular endothelial growth factor-C (VEGF-C), leading to inhibition of tumor progression [10]. Many novel checkpoint proteins have been described, and divergent effects have been reported for some of these markers, such as the immunomodulator B7-H3 (CD276), with immunosuppression and pro-tumorigenic activity on the one hand, but stimulation of CD4+ and CD8+ lymphocyte activity on the other. Nevertheless, CD276 has been confirmed to be overexpressed in many tumor types, and the prevailing opinion is that CD276 supports glioma progression and should be considered as an important target for glioma therapy, which is also true for natural killer receptor protein 1 (CD161) overexpressed in gliomas [13,14,21]. In recent years, five different hypofunctional states of CD8+ T cells in tumors have been described and are currently being explored in different tumor types: senescence, anergy, exhaustion, tolerance and ignorance. The common feature of these states is an altered immunological T cell response due to various reasons, such as the shortening of telomeres (T cell senescence) or repeated antigen exposure under suboptimal conditions (T cell exhaustion). Currently, the prevailing view is that all of these pathological CD8+ T cell states occur within gliomas and show regional variation during tumor progression. Furthermore, the fundamental question arises whether these five different stages can be clearly delineated from each other, or whether, for example, the phenomenon of T cell anergy should be subsumed under the term T cell exhaustion [1,2,25,29,31]. The understanding of the importance of B cells has also expanded, an important example being the description of a subset of B cells with CD137+ expression, which is a cellular subset with an antitumor effect due to stimulation of CD8+ cytotoxic T cells (Table 2) [35,36]. One of the most important findings concerning TAMs is that their previous classification into the two categories of pro-inflammatory M1 type and immunosuppressive and pro-tumorigenic M2 type is still a valid classification in terms of macrophage functionality, but at the molecular level, additional subtypes of TAMs have been described [2,39,40,50]. One example is the description of a subtype with expression of CD169+ and pro-inflammatory cytokines leading to the recruitment and activation of effector T cells and NK cells. The antitumor activity of CD169+ expression was confirmed by its experimental silencing, which resulted in shorter animal survival [55]. In gliomas with PTEN mutations, a large number of immunosuppressive macrophages were detected in the glioma tissue. This is a remarkable result because PTEN is the most frequently inactivated tumor suppressor in primary gliomas, which is clearly linked by this result to the number of TAMs as one of the key criteria in assessing the tumor environment [60]. An important recent finding is the demonstration of the existence of “double-positive TAMs”, which simultaneously exhibit the molecular signature of macrophages and glioma tumor cells. In vitro analyses confirmed that these cells can be formed by macrophages via phagocytosis of tumor cells, and furthermore, their existence was confirmed in the tumors of glioblastoma patients. Double-positive TAMs exhibit immunosuppressive properties through the expression of checkpoint proteins such as PD-L1 and CD276. This finding provides a deeper understanding of the phenomenon of phagocytic macrophage-mediated immunosuppression in gliomas, which ultimately leads to decreased treatment efficacy [62]. Myeloid-derived suppressor cells (MDSCs) have the ability to suppress the antitumor activity of NK cells and CD4+ and CD8+ T cells in the TEM of gliomas. The two major groups of MDSCs are derived from granulocytes (G-MDSCs) and from monocytes (M-MDSCs), but additional molecular subtypes of MDSCs have since been described [63,64,65]. Several reasons exist for the tumor-promoting properties of MDSCs in gliomas, and in addition to the expression of checkpoint proteins such as PD-L1, increased expression of arginase, integrin ß1 and dipeptidyl peptidase 4 (DPP-4) has also been described [2,63,66,68]. An important recent observation regarding MDSCs in gliomas is the demonstration of high expression of vascular non-inflammatory molecule 2 (vanin-2, VNN2) by monocyte-derived M-MDSCs in the peripheral blood of healthy individuals. In patients with gliomas, the expression of VNN2 in M-MDSCs in peripheral blood was significantly reduced compared with healthy controls, and the degree of reduction in VNN2 expression was inversely correlated with glioma grade. This finding suggests that peripheral monocyte-derived M-MDSCs expressing CD14+/VNN2^high^ represent a population of MDSCs unique to healthy subjects. The reason for the reduced VNN2 expression in the biological response of M-MDSCs to gliomas has not yet been investigated, but the detection of CD14+/VNN2^high^ MDSCs in peripheral blood is considered an additional useful marker in the diagnostic spectrum of tumor diagnosis [73].

**Table 2:** Cellular components of the tumor environment (TEM) in gliomas—important new aspects.

**Lymphocytes:** Evidence of migration of CD8+ T cells from deep cervical lymph nodes into gliomas in a *mouse* model after stimulation with vascular endothelial growth factor-C (VEGF-C), resulting in rapid inhibition of tumor growth [10]. Confirmation of increased expression of natural killer receptor protein 1 (CD161) on glioma-infiltrating cytotoxic CD8-positive T cells in high-grade and in low-grade gliomas, leading to inhibition of T cell activity; CD161 expression inversely correlates with prognosis [21]. Increasing realization of the importance of B lymphocytes as part of the TEM in gliomas; evidence of distinct formation of tertiary lymphoid structures during tumor progression with B cells as major component; recognition of a B cell subtype with CD137+ expression showing antitumor activity due to activation of cytotoxic CD8+ T cells [35,36].
**Microglia and myeloid-derived cells:** Description of a subset of tumor-associated macrophages (TAMs) characterized by CD169+ expression that produce pro-inflammatory cytokines, leading to T cell and NK cell activation. Experimental depletion of CD169+ macrophages results in shorter survival of glioma *mice* [55]. Investigation of the so-called “double-positive TAMs” with expression of the molecular signature of glioma cells and macrophages. These cells originate from phagocytosis of glioma cells by TAMs and are a major factor in TAM-mediated immunosuppression in gliomas [62]. There is evidence of downregulation of the surface ectoenzyme vanin-2 (VNN2) on monocytic myeloid-derived suppressor cells (M-MDSCs) in the peripheral blood of patients with high-grade gliomas compared with the peripheral blood of healthy individuals. The need for inclusion of VNN2 in the arsenal of M-MDSC markers is addressed [73].
**Glioma stem cells:** Glioma stem cells do not represent a uniform state during tumor progression but undergo developmental programs that lead to changes in their phenotype [74,88]. Detection of partially overlapping features of temozolomide-chemoresistant glioma tumor cells and glioma stem cells after exposure of parental glioma cells to temozolomide (TMZ) in vitro; most tumor cells in daughter colonies were chemoresistant and showed expression of stem cell markers CD133 and nestin; a minority of daughter cells remained chemosensitive [94]. Evidence for the influence of transcription factors associated with circadian rhythm on the maintenance of glioma stem cells and angiogenesis [101,102,103].
**Neutrophils:** Significantly higher numbers of neutrophils in glioblastomas with mutation of the gene for the enzyme telomerase reverse transcriptase (TERT) compared to TERT wild-type tumors; neutrophils in gliomas with TERT mutation express large amounts of chemokines responsible for neutrophil settlement in the tumor and tumor progression [106,108].
**Cancer-associated fibroblasts:** Confirmation of the presence of cancer-associated fibroblasts (CAFs) in glioblastomas with proximity to glioma stem cells, endothelial cells and macrophages; significantly shorter overall survival of patients with glioblastomas showing high intratumoral expression of the CAF marker actin alpha 2 (ACTA2) [113].
**Dendritic cells:** Detailed molecular analysis of dendritic cells (DCs) in IDH-mutated and IDH wild-type gliomas; confirmation of the occurrence of DCs with delayed maturation in IDH-mutated gliomas due to the paracrine influence of 2-R-hydroxyglutarate; the presence of fully functional DCs in late-stage gliomas with IDH wild-type [119]. Evidence of thyroid-stimulating hormone (TSH) expression by DCs in gliomas; TSH promotes tumor progression and PD-L1 expression with immune evasion in gliomas with high TSH receptor (TSHR) expression; DCs represent the primary source of TSH in gliomas [120].
**Non-neoplastic neuronal and glial cells:** A deeper insight into the dysregulation of the interaction between glutamatergic neurons and γ-aminobutyric acid containing neurons (GABAergic neurons) in the TEM of gliomas; the decreased electrophysiological activity of parvalbumin-containing “fast-spiking” GABAergic interneurons appears to play a central role in this dysregulation [121,122,123,124,125,126]. New insights into the intercellular relationships between glioma cells and non-neoplastic astrocytes through extracellular vesicles, gap junctions and tunneling nanotubes [135,136,137,138,139,140,141,142,143].

Based on recent in vitro studies and observations in human gliomas, glioma stem cells are no longer considered as a unique state during tumor progression, but as a cell type that undergoes phenotypic changes in terms of cellular and molecular properties. One of the most important recent findings in this context is the significant association of different cellular phenotypes with distinct expression profiles for stem cell markers [74,88,89]. There is also emerging evidence on spatial tumor architecture in relation to the localization of different stem cell phenotypes in high-grade gliomas, as stem cells with an oligodendrocyte progenitor-like phenotype are predominantly localized in the tumor core, whereas radial glial stem cell-like cells are found at the tumor margin [91]. Another important recent finding is an association between glioma stem cells and the phenomenon of resistance to temozolomide (TMZ) treatment. In vitro exposure of glioma tumor cells to TMZ generated cellular daughter colonies that contained TMZ-resistant tumor cells with additional expression of stem cell markers. This finding should be considered extremely important because it confirms that the phenotypes of chemoresistant glioma tumor cells and glioma stem cells appear to overlap, which may explain, at least in part, the emergence of TMZ-resistant tumor cells after the initial exposure of their parent cells to TMZ [94]. Transcription factors involved in the interdependence between cellular functions and circadian rhythm are of growing scientific interest, and their continuous activity has been shown to be a prerequisite for the maintenance of tumor progression and stem cell activity in various tumor types. Some of these transcription factors are known to promote transcription, while others act as suppressors of transcriptional activity. The CLOCK-BMAL1 complex, which is a complex of two transcription factors, promotes angiogenesis in malignant gliomas by upregulating the extracellular matrix protein periostin (POSTN). Since tumor progression and angiogenesis could be significantly inhibited by experimentally blocking transcription factors associated with circadian rhythm, they are further investigated as potential therapeutic targets [101,102,103].

Neutrophils, mast cells and cancer-associated fibroblasts are considered as cell types involved in promoting glioma progression. The number of neutrophils in TEM correlates with the tumor grade of gliomas and inversely with the prognosis of patients. In particular, for glioblastomas, a significantly higher number of neutrophils was confirmed in cases with TERT mutation. In the cases with TERT mutation, neutrophils showed significantly higher expression of cytokines, resulting in significant homing of more neutrophils to the tumor and further tumor progression [106,108]. The number of mast cells in TEM also correlates with tumor grade, and their degranulation within the tumor leads to the release of chemokines and other tumor-promoting compounds such as CXCR4, STAT3 and serglycin which trigger further tumor progression [110,111]. Cancer-associated fibroblasts in gliomas are enriched in perivascular regions and show proximity to endothelial cells, macrophages and glioma stem cells. An important recent finding confirming the tumor-supportive role of CAFs is the strong expression of the CAF marker actin alpha 2 (ACTA2) in glioblastomas, and the amount of ACTA2 expression inversely correlates with overall patient survival [113]. Dendritic cells (DCs) are not present in healthy human brain parenchyma, but they are abundant in gliomas. Differences were demonstrated between gliomas with and without IDH mutation, as transmigrated DCs exhibited delayed maturation due to the paracrine activity of 2-R-hydroxyglutarate in IDH-mutated gliomas, resulting in decreased expression of interleukin-6 and MHC-mediated antigen presentation. This finding is considered prognostically relevant and the dysfunctional DCs in IDH-mutated gliomas are considered a potential target for immunotherapy [119]. This is also true for thyroid-stimulating hormone (TSH) detection and evidence that DCs are the major source of TSH within TEM in gliomas. TSH promotes the expression of the checkpoint protein PD-L1 and promotes proliferation and further tumor growth, which has been confirmed for several tumor types with high expression of the TSH receptors (TSHR), including gliomas. Therefore, the TSHR is also considered as a potential target for immunotherapeutic approaches for the treatment of gliomas [120].

Non-neoplastic neuronal cells in TEM contribute to glioma progression through the expression of neuronal mitogens such as neuroligin3, brain-derived neurotrophic factor and semphorin-4F [123,124]. Even the upregulation of glutamate and increased expression of glutamate receptors must be considered as tumor-promoting conditions that occur at the tumor margin where many non-neoplastic neurons are still present. A third important factor contributing to glioma progression is deregulation of the neuronal GABAergic system, which is particularly true for “fast-spiking” interneurons. Several factors contribute to this dysregulation of the GABAergic system, such as hyperactivated mTOR signaling or decreased expression of potassium chloride cotransporter 2. According to these recent findings in non-neoplastic neurons in TEM, it can be considered certain that the impaired interplay between excitatory and inhibitory neurons contributes to tumor progression [123,124,125,126,127]. Non-neoplastic astrocytes also contribute to glioma progression by expressing tumor-promoting proteins such as TGF-ß, STAT3, CCL2 and CSF1 in later tumor stages after causing initial astrogliosis at the tumor boundary in earlier tumor stages [135,136,137]. Of greatest importance is the fact that of all cell types in TEM of gliomas, astrocytes exhibit marked intercellular communication with glioma cells via transport through extracellular vesicles (EVs), gap junctions and nanotubes, and the distinct effects of this intercellular communication on glioma progression have been confirmed. Exposure of astrocytes to EVs derived from glioma cells leads to increased migratory activity of astrocytes with increased expression of cytokines that promote proliferation and tumor growth [138]. Rapid formation of gap junctions between glioma cells and astrocytes has been demonstrated in vitro, and the major gap junction protein, connexin 43, is significantly associated with increased glioma progression and unfavorable patient prognosis [139,140,141]. Tunneling nanotubes (TNs) have been studied for many years, but in particular, the study of TNs between astrocytes and tumor cells and their influence on the biological behavior of gliomas is still in its infancy. In a co-culture system of glioma cells and astrocytes, a significant reduction in the proliferation potential of glioma cells was observed due to the formation of nanotubes [142]. Other studies suggest that TNs could be used by cancer cells to adapt astrocytes to hypoxic and metabolic tumor conditions [143]. An important aspect in this context is the transport of non-coding RNA molecules (ncRNAs) through this system of intercellular junctions, with micro-RNAs (miRNAs) being the most studied ncRNAs to date. The entire topic of epigenetic processes in gliomas attributable to the activity of ncRNAs is vast and far beyond the scope of this review of the tumor environment. An important example in brain tumors is the downregulation of PTEN expression in brain tumor metastases through the introduction of multiple PTEN-targeting miRNAs by astrocyte-derived EVs [147]. Another important observation is that miRNAs cause inhibition of the mTOR pathway in gliomas, leading to attenuation of glioma cell migration and invasion. These miRNAs are downregulated in EVs released from gliomas by immunosuppressive macrophages [148]. There is a consensus in the literature that these systems of intercellular communication open up far-reaching prospects for effective therapeutic delivery of molecules including proteins and nucleic acids [149,150].

Treatment strategies for gliomas as well as related experimental efforts and in vitro studies are a topic in itself, with some research approaches focusing on the cellular components of TEM. The current standard of care for glioblastoma is still surgical resection, adjuvant radiotherapy and chemotherapy with the alkylating agent temozolomide (TMZ). Several factors can attenuate the cytotoxic effect of TMZ therapy. One known factor is the lack of methylation of methyl-guanine methyltransferase (MGMT), which leads to protection of glioma cells due to DNA repair. As addressed in the present review, elements of TEM also contribute to TMZ resistance, such as immunosuppressive TAMs due to activation of the STAT3-MYC signaling pathway and the presence of gap junctional communication between glioma cells and non-neoplastic astrocytes [51,53,54,140,141]. In terms of molecular therapeutic approaches for gliomas, two therapeutic approaches are already standard of care for patients with gliomas harboring BRAF-V600E alterations and for patients with tuberous sclerosis and subependymal giant cell astrocytoma (SEGA). According to the European Association of Neuro-Oncology (EANO), treatment with RAF-kinase inhibitors such as dabrafenib is recommended for patients with progressive gliomas that have BRAF-V600E alterations. The use of mTOR inhibitors in patients with tuberous sclerosis and SEGA also significantly reduces seizure frequency, and therefore, the use of mTOR inhibitors such as everolimus is being considered as part of standard treatment when surgical removal of SEGA is not possible [151]. Molecular therapy trials involving cellular elements of TEM are still under investigation. Various approaches have been described, with the investigation of small molecule inhibitors and immunomodulatory agents representing two main areas of interest. The term “small molecules” (SMs) refers to organic compounds of low molecular weight as opposed to larger molecules such as proteins or nucleic acids. Many SMs exhibit better blood–brain barrier (BBB) permeability, and delivery through nanoparticles is a rewarding process under investigation in experimental and in vitro conditions [152,153]. An important example of the targeting of elements of the TEM by SMs is the small molecule gambogic amide (GA-amide), which shows good penetration through the BBB. GA-amide promotes the formation of a protein complex involving WD repeat domain 1 (WDR1) and cofilin, leading to the inhibition of migration and tumor-sphere formation involving tumor cells and glioma stem cells [154]. Another example is the delivery of two SMs by nanoparticles targeting the canonical and non-canonical NF-kB signaling pathway in myeloid cells from TEM. This experimental approach resulted in increased expression of interleukin 12 (Il-12) in myeloid cells with increased activity of T effector cells and decreased the tumor growth of gliomas [152].

Recent reports on clinical therapy trials targeting immune cells from the TEM show a broad spectrum of immunological approaches (Table 3). The use of dendritic cell vaccines is one of the most common methods for many tumor types to mobilize a broad immune response, including mobilization and activation of effector T cells. For patients with gliomas, vaccines derived from autologous and allogeneic dendritic cells have been reported to be well tolerated without serious side effects and to achieve significantly longer median overall patient survival compared with patients receiving standard treatment [155,156]. Chimeric antigen receptor T cell therapy is another common approach to immunotherapy. In a phase I trial, four patients underwent CAR-T cell therapy targeting disialoganglioside G2 present in H3K27M-mutated gliomas. Because of its specificity for tumors such as melanoma, neuroblastoma or H3K27M-mutated glioma, G2 is considered a suitable target for immunotherapy of these tumor types. Three of the four treated patients with H3K27M-mutated gliomas experienced clinical and radiological improvement without serious side effects [157]. Other therapeutic targets included the cancer-associated protein survivin, which is known to inhibit T cell activation in tumors, and the inhibitor of the Nod-like receptor family pyrin domain containing 3 (NLRP3), which is predominantly expressed in macrophages and contributes to their immunosuppressive function in tumors. In the study using an antibody against survivin, treated patients with glioblastoma had a median overall survival of 25.9 months [158]. In the study using an antibody against NLRP3, 3 of 16 patients had an overall response to treatment [159]. Human recombinant bone morphogenetic protein 4 (hrBMP4) was used to treat 15 patients with glioblastoma recurrence after chemoradiation therapy. Three patients showed a response, including two patients with a complete response 30 and 57 months after hrBMP4 administration [160]. A special trial performed for the first time in *humans* is the administration of the herpes simplex virus *thymidine kinase suicide gene* (*HSV-TK*) using an adipose-derived mesenchymal stem cell vehicle for delivery. In principle, suicide gene therapy leads to the formation of toxic metabolites from pro-drugs such as ganciclovir, which in turn lead to intratumoral inhibition of DNA polymerase and cell death. Twelve patients with glioblastoma who did not undergo surgery after recurrence were treated. Eight patients showed a partial response or stable disease. A significant increase in the number of T cells was confirmed during therapy [161]. A marked immunologic response with T cell response and increased IFN titers was also obtained in six patients with recurrent glioblastoma treated by inoculation with the oncolytic herpes simplex virus G207. Two patients were long-term survivors, and no serious side effects of therapy were reported. Of note, the patient with the longest survival of 7.5 years had the highest expression levels for several T cell-related genes [162]. Most of these clinical trials reported in the last two years are phase I trials, but based on the reports of long-term survivors and good therapeutic tolerability without serious side effects for most trials, there is strong evidence that therapeutic targeting of cellular components of the tumor environment is useful and promising in gliomas. In summary, our review has shown that many new insights into the cellular components of the tumor environment of gliomas have been gained in recent years. These new insights concern the biology of individual cell types, their interplay and the influence of such factors on tumor progression that have received little or only marginal attention in glioma research. Good examples are the phenomenon of double-positive tumor-associated macrophages or the influence of thyroid-stimulating hormone (TSH) on the progression of gliomas. Overall, there is a consensus that the cellular components of the tumor environment are as important for understanding the biological behavior of gliomas as the signaling pathways of the tumor cells themselves, which is also important for designing further approaches to immunotherapy. In this context, targeting components of the TEM represent a major methodological aspect with great potential in order to explore new and effective therapeutic strategies for patients with gliomas.

## Figures and Tables

**Table 1:** Overview of the cellular components of the tumor environment (TEM) in gliomas and their main pathophysiological changes.

Lymphocytes	Dysregulation due to inhibition of CD8+ effector cells and natural killer cells via different pathways.
Microglia	Brain resident cells showing molecular differences compared with bone marrow-derived macrophages. Together, these cell types are subsumed under the term “Glioma-associated macrophages” (GAMs). There are different views concerning the exact pathophysiological role of microglia in gliomas.
Macrophages	Bone marrow-derived cells invading the glioma tissue and termed “Tumor-associated macrophages” (TAMs) together with microglia. The majority of TAMs are in an immunosuppressive state, promoting tumor growth.
Myeloid-derived suppressor cells	Immature myeloid cells invading glioma tissue and promoting tumor growth through various pathways.
Glioma stem cells	Tumor cells expressing typical stem cell markers with the ability to produce new and phenotypically distinct cancer stem cell clones capable of promoting tumor progression.
Neutrophil granulocytes	This cell type often shows high abundance in glioma tissue with a large number of neutrophils, indicating a more aggressive behavior of the tumor.
Mast cells	Degranulation of mast cells with the release of chemokines triggers tumor infiltration of other immune cells and tumor progression.
Cancer-associated fibroblasts	Present in glioma tissue with pro-tumoral effects due to the upregulation of tumor-promoting pathways.
Dendritic cells	Not present in the normal brain parenchyma. Evidence of a defective differentiation in gliomas leading to the promotion of tumor growth.
Neurons	Dysregulation of transmitter release and expression of pro-tumorigenic factors.
Oligodendrocytes	Only a few data concerning the role of non-neoplastic oligodendrocytes in gliomas.
Astrocytes	Distinct intercellular relationship with glioma cells through various tumor-promoting molecular pathways and exchange of extracellular vesicles.

**Table 3:** Important examples of current immunotherapeutic clinical trials reported in 2022–2023 targeting cellular components of the tumor environment in glioma. GBM = glioblastoma; A = astrocytoma; mOS = median overall survival; ST = standard treatment; CAR-T cells = chimeric antigen receptor T cells.

First Author, Year, Phase of Study	Therapeutic Agent	Type of the Agent
Liau, 2023, Phase III [155]	DC-Vax-L	Autologous tumor lysate-loaded dendritic cells
Significantly longer mOS of patients with newly diagnosed or relapsed GBM compared to patients receiving ST. Significant differences for the group with relapsed GBM (13.2 vs. 7.8 months) and for the group with MGMT methylation (30.2 vs. 21.3 months). The vaccine was well tolerated.		
Lepski, 2023, Phase II [156]	No definite name of the vaccine so far	Allogenic dendritic cells
Treatment of patients with GBM or A (WHO grade 4) at the time of first relapse after surgery. Significantly longer mOS for patients with GBM compared with patients who received ST (27.6 vs. 16.3 months). Significantly longer mOS for patients with A (grade 4) (59.5 vs. 19.8 months). No adverse effects were observed, although one patient had mild and transient hepatitis during treatment.		
Majzner, 2022, Phase I [157]	No definite name of the vaccine so far	Autologous GD2-CAR T cells
CAR-T cell vaccine directed against disialoganglioside G2 expressed in H3K27M-mutated glioma cells. Treatment of the first four patients (age 5–25 years) with H3K27M-mutated midline and spinal cord gliomas. Clinical and radiographic improvement occurred in three of the four patients. No evidence of treatment-related adverse effects was found.		
Ahluwalia, 2022, Phase II [158]	SurVaxM	Peptide directed against survivin
Treatment of patients with GBM: mOS = 25.9 mo, and clinical benefit was observed in patients with and without MGMT methylation. Evidence of increased CD8+ T cell response during treatment was found. No adverse effects were observed except localized granulomatous panniculitis in two patients.		
Fine, 2023, Phase I, [159]	RRx-001	Inhibitor of the Nod-like receptor family pyrin domain containing 3 (NLRP3)
In 16 patients with newly diagnosed GBM, RRx-001 therapy in combination with ST did not result in serious adverse events, and life expectancy was 21.9 months. Three patients had an overall response to treatment. Stable disease or even modest improvement was observed in 11 patients.		
Bos, 2023, Phase I [160]	Human recombinant bone morphogenetic protein 4 (hrBMP4)	Inhibitory regulator of cancer stem cells
A total of 15 GBM patients with relapse after chemo-radiotherapy. One patient had a partial response and two patients had a complete tumor response that lasted until the last follow-up, 57 months and 30 months, respectively, after hrBMP4 administration. The therapy was well tolerated and had no serious adverse effects.		
Oraee-Yazdani, 2023, Phase I [161]	HSV-TK	Allogenic mesenchymal stem cells carrying the herpes simplex virus-thymidine kinase suicide gene
GBM patients who had not undergone surgery for relapse. The mOS was 16.0 months, with two-thirds of patients showing a partial response and stable disease. No adverse effects attributable to the study intervention were reported.		
Miller, 2022, Phase I, [162]	G207-oncolytic HSV	Oncolytic herpes simplex virus G207
Six GBM patients with relapse; a retrospective analysis of tumor tissue after phase I trial. Two of the six patients were long-term survivors (5.5 and 7.5 years), and no serious side effects of therapy were reported. Immunologic expression analysis of tumor tissue revealed a marked antiviral response, including chemotaxis of immune cells, which was most pronounced in the patient with the longest survival.		

## Data Availability

Not applicable.

## Abbreviations

**α-SMA:** smooth muscle actin α; **ACTA2:** actin alpha 2; **ALDH1:** aldehyde dehydrogenase 1; **AP-1:** activator protein 1; **BDNF:** brain-derived neurotrophic factor; **BMAL1:** brain and muscle ARNT like protein 1; ***BRAF:*** *rapid accelerated fibrosarcoma B gene*; **C1QL1:** complement component 1 q subcomponent-like 1; **CAFs:** cancer-associated fibroblasts; **CAR-T cell:** chimeric antigen receptor T cells; **CCL5:** chemokine C-C ligand 5; **CCL22:** chemokine C-C ligand 22; **CD 276:** immune-modulator B7-H3; **CD161:** natural killer receptor protein 1; **CD4+ cells:** helper T lymphocytes with expression of CD4; **CD8+ cells:** cytotoxic (“effector”) T lymphocytes with expression of CD8; **CDKN2A/B:** cyclin-dependent kinase inhibitor 2A/B; **CHI3L1:** chitinase-3-like protein 1; **CLOCK:** circadian locomotor output cycles kaput; **CRY:** cryptochrome circadian regulator; **CSCL9/10:** C-X-C motif chemokine ligand 9/10; **CSF1:** macrophage colony-stimulating factor 1; **CX3CR1:** CX3C motif chemokine receptor 1; **DCs:** dendritic cells; **DHX9:** DEAD/DEAH box helicase 9; **DUSP3:** dual-specificity phosphatase 3; **EANO:** European Association of Neuro-Oncology; **EGFR:** epidermal growth factor receptor; **ELISA:** enzyme-linked immunosorbent assay; **ERK:** extracellular signal-regulated kinase; **EVs:** extracellular vesicles; **FAM20C:** family with sequence similarity 20, member C; **Fas-L:** Fas-ligand; **FCGR2B:** Fc Fragment of IgG—low Affinity II—Receptor for CD32B; **FGL2:** fibrinogen like protein 2; **G6PD:** glucose-6-phosphatase dehydrogenase; **GABA:** γ-aminobutyric acid; **GAMs:** glioma-associated microglia/macrophages; **GDNF:** glial cell-derived neurotrophic factor; **GFAP:** glial fibrillary acid protein; **Glut:** glutamate; **GPD1:** glycerol-3 phosphate dehydrogenase 1; **GSK3β:** glycogen synthase kinase 3-β; **H3K27M mutation:** mutation of histone3 at position 27 with exchange of methionine by lysine; **HA:** hyaluronic acid; **HDGF:** hepatocyte-derived growth factor; **HIF-1α:** hypoxia inducible factor 1α; **HLA-DR:** human leukocyte antigen—DR isotype; **hrBMP4:** human recombinant bone morphogenetic protein 4; ***HSV-*TK**: *thymidine kinase suicide gene*; **IDO1:** tryptophan-degrading enzyme indoleamine 2, 3-dioxygenase; **IDH:** isocitrate dehydrogenase; **IFN-γ:** Interferon γ; **Il-10:** interleukin 10; **JUN:** Jun N-terminal kinase; **KCC2:** potassium-chloride-cotransporter 2; ***KLRB1:*** *Killer Cell Lectin Like Receptor B1 gene*; **Kv1.3:** voltage-gated potassium-channel protein Kv1.3; **LAG-3:** lymphocyte-activation 3; **LDHA:** lactate dehydrogenase A; **LRP-1:** low-density lipoprotein receptor-related protein 1; **MAPK:** mitogen-activated protein kinase protein family; **MDM2:** mouse double minute 2 homolog; **MDSCs:** myeloid-derived suppressor cells; **MGMT:** methyl-guanine methyltransferase; **MHC1:** major histocompatibility complex class 1; **miRNAs:** micro RNAs; **MMP2:** metalloproteinase 2; **MYC:** avian myelocytomatosis viral oncogene; **ncRNAs:** non-coding RNA-molecules; **NCX:** Na^+^/Ca^2+^ exchanger; **NF1:** Neurofibromin 1; **NF2:** Neurofibromin 2; **NFAT:** nuclear factor of activated T cells; **NF_K_B:** nuclear factor kappa-light-chain enhancer of activated B cells; **NKG2D:** natural killer group 2D; **NKs:** natural killer cells; **NLGN3:** neuroligin3; **NLRP3:** Nod-like receptor family pyrin domain containing 3; **P2RY12:** purinergic receptor P2Y12; **PAI-1:** Plasminogen activator inhibitor 1; **PDGFR:** platelet-derived growth factor receptor; **PD-L1:** programmed death receptor ligand-1; **PER:** periodic circadian protein homolog 1; **POSTN:** periostin; **PTEN:** phosphatase and tensin homolog; **PTPRZ1:** protein tyrosine phosphatase receptor zeta 1; **RAS:** rat sarcoma protein family; **ROS:** reactive oxygen species; **RUNX1:** runt-related transcription factor 1; **S100A4:** S100 calcium binding protein A4; **Sall1:** spalt-like transcription factor 1; **SEGA:** subependymal giant cell astrocytoma; **SEMA4F:** transmembrane protein semaphorin-4F; **SM:** small molecule; **SSEA-1:** Stage-specific embryonic antigen-1; **STAT3:** signal transducer and activator of transcription 3; **STI1:** stress-inducible protein 1; **TAMs:** tumor-associated macrophages; **TBK1:** TANK-binding kinase 1; **TEM:** tumor environment; **TERT:** telomerase reverse transcriptase; **TGF-ß:** transforming growth factor ß; **TIM-3:** T cell immunoglobulin and mucin-domain containing-3; **TLR2:** toll-like receptor 2; **TLS:** tertiary lymphoid structures; **Tmem119:** transmembrane protein 119; **TMZ:** temozolomide; **TNs:** tunneling nanotubes; **TOX:** thymocyte selection-associated high-mobility group box protein; **TP53:** tumor protein 53; **Tregs:** T regulatory cells; **TSH:** thyroid-stimulating hormone; **TSHR:** thyroid-stimulating hormone receptor; **tTG:** tissue transglutaminase; **VEGF-C:** vascular endothelial growth factor C; **VNN2:** surface-ectoenzyme vanin-2; **WDR1:** WD repeat domain 1; **WIF1:** Wnt inhibitory factor 1; **WNT5A:** wingless-related integration site family member 5A; **xCT:** cystine glutamate antiporter; **Zap70:** linker of activated T cells and phospholipase Cγ1.
